# Functionalization of Cyclodextrins with *N*-Hydroxyphthalimide Moiety: A New Class of Supramolecular Pro-Oxidant Organocatalysts

**DOI:** 10.3390/molecules200915881

**Published:** 2015-08-31

**Authors:** Lucio Melone, Manuel Petroselli, Nadia Pastori, Carlo Punta

**Affiliations:** 1 Department of Chemistry, Materials, and Chemical Engineering “G. Natta”—Politecnico di Milano, Piazza Leonardo da Vinci 32, Milano I-20133, Italy; E-Mails: manuel.petroselli@polimi.it (M.P.); nadia.pastori@polimi.it (N.P.); carlo.punta@polimi.it (C.P.); 2 INSTM, National Consortium of Materials Science and Technology, Local Unit Politecnico di Milano, Milano 20133, Italy; 3 Università Telematica e-Campus, Via Isimbardi 10, Novedrate 22060, Italy

**Keywords:** *N*-hydroxyphthalimide, cyclodextrin, polyunsaturated fatty acids, lipid peroxidation, reactive oxygen species, oxidative stress, cancer

## Abstract

*N*-hydroxyphthalimide (NHPI) is an organocatalyst for free-radical processes able to promote the aerobic oxidation of a wide range of organic substrates. In particular, NHPI can catalyze the hydroperoxidation of polyunsaturated fatty acids (PUFA). This property could be of interest for biological applications. This work reports the synthesis of two β-cyclodextrin derivatives (**CD5** and **CD6**) having a different degree of methylation and bearing a NHPI moiety. These compounds, having different solubility in water, have been successfully tested for the hydroperoxidation of methyl linoleate, chosen as the PUFA model molecule.

## 1. Introduction

Reactive oxygen species (ROS) are free radicals (FR) deriving from oxygen, produced as a normal part of metabolism within the mitochondria. External factors (*i.e.*, smoking, UV-light exposition or environmental pollutants) can stimulate ROS production [1,2]. Increased levels of ROS may induce oxidative stress (OS), damaging proteins, DNA and RNA molecules, sugars and lipids, thus affecting negatively the cells structure and their functions. Different chronic diseases, such as Alzheimer’s disease, diabetes mellitus, hypertension, and cancer in both animals and humans, are associated to the negative effects of the ROS cellular overproduction. With regard to cancer, there is a considerable body of experimental evidence on the tumorigenesis caused by the alteration of the redox status of the cells [3,4]. In order to reduce the OS, several antioxidants have been proposed as ROS scavengers [5], even if not all the studies pointed in the same direction regarding the beneficial effects of antioxidants to prevent the development of cancer [6,7]. A different strategy proposed in the last few years for the treatment of tumors relies on the promotion of the OS instead of its inhibition. In fact, compared to normal cells, cancer cells exhibit higher ROS levels, but they are also more sensitive to further increments of OS [8]. In other words, promoting the ROS production in tumoral tissues up to cytotoxic levels would selectively induce apoptosis of the cancer cells [9,10]. The development of new pro-oxidant molecules able to enhance the OS of cancer cells has, therefore, relevant scientific interest.

**Scheme 1:**
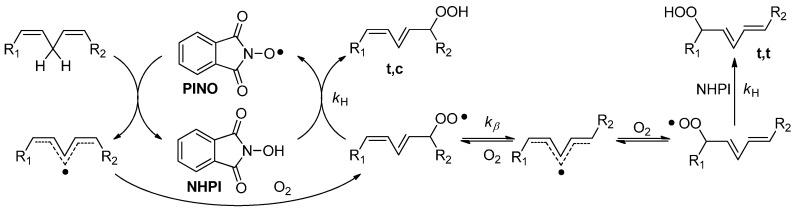
NHPI-mediated peroxidation of a generic bis-allylic derivative.

*N*-hydroxyphthalimide (NHPI) is an emerging organocatalyst capable of promoting free-radical aerobic oxidations by activating the hydrogen atom transfer processes via *in situ* generation of phthalimide-*N*-oxyl (PINO) radical (Scheme 1) [11,12,13,14,15]. NHPI has been extensively used for the oxidation of different organic compounds and has industrial relevance [16,17,18,19,20,21,22]. Due to its capability of catalyzing the peroxidation of lipids [23], NHPI could play a role in biomedicine as a pro-oxidant agent for cancer therapy. Unfortunately, the NHPI derivatives proposed in the literature for industrial use have been synthetized to be dissolved in liquid hydrocarbons, eventually using a co-solvent to increase their solubility [24,25]. Therefore, they are not suitable to be tested for the envisaged biological application. The synthesis of water-soluble derivatives bearing NHPI moieties could represent a valuable solution to tackle this problem. Moreover, the suitable choice of functional groups to be introduced into NHPI should also favor penetration of the cellular membrane, allowing the catalyst to manifest its pro-oxidant activity in the intracellular medium. Methyl-β-cyclodextrin (MeβCD), is a highly water soluble cyclic heptasaccharide that has been proposed in cancer therapy due to its capability to deplete cholesterol from the membrane cells, thus enhancing the effects of anticancer drugs [26,27,28]. In the present work we describe the synthesis of two NHPI-functionalized MeβCD derivatives, having different degrees of methylation, and we investigate their activities in the aerobic peroxidation of methyl linoleate, chosen as model molecule among the several polyunsaturated fatty acids (PUFA). Due to the synergistic action of the NHPI moieties, working as promoter of the oxidative stress, and MeβCD acting as lipid raft disrupting agent, the proposed supramolecular vectors could represent potential pro-oxidant agents in biological systems.

## 2. Results and Discussion

### 2.1. Synthesis and Characterization of NHPI-Functionalized MeβCD Derivatives

Cyclodextrins (CDs) are macrocyclic oligosaccharides composed of 6, 7, or 8 α-[1–4]-linked α-d-glucopyranose units (α-, β- and γ-CD, respectively). These molecules have a truncated cone-shaped three-dimensional structure with an empty, hydrophobic, inner cavity. The hydroxyl groups of the anhydroglucose units are located both on the large rim (OH in position 2 and 3) and on the small rim of the truncated cone (OH in position 6). Among the three CDs, βCD is the cheapest and most used, despite the fact that its solubility in water at 25 °C (18.5 mg·mL^−1^) is lower than that of αCD (145 mg·mL^−1^) and γCD (232 mg·mL^−1^). The random methylation of the βCD hydroxyl groups leads to derivatives that are more soluble. Indeed, commercially available MeβCD, with a degree of substitution (DS, average number of methyl groups per glucose repeat unit) equal to 1.8, has a very high solubility in water (>500 mg·L^−1^ at 25 °C). MeβCD has found several applications in the last few years, spanning from pharmaceutics and health care [29] to soil remediation [30]. The capability of MeβCD to act as disruptor of lipid rafts selectively binding cholesterol is well documented [31], thus, offering a new tool for cancer therapy [26,27,28].

**Scheme 2:**
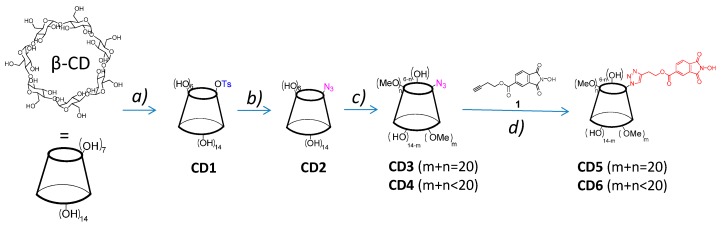
Synthetic scheme. (**a**) TsCl, NaOH(aq) 0.4 M, 0 °C 8 h; (**b**) NaN_3_, DMSO, 90 °C, 12 h; (**c**) dry DMF, NaH, CH_3_I, 0 °C, 8 h; (**d**) THF:H_2_O = 1:1 (*v*:*v*), **1**, l-ascorbic acid, Cu^2+^, 40 °C, 24 h, N_2_ atmosphere.

**Figure 1:**
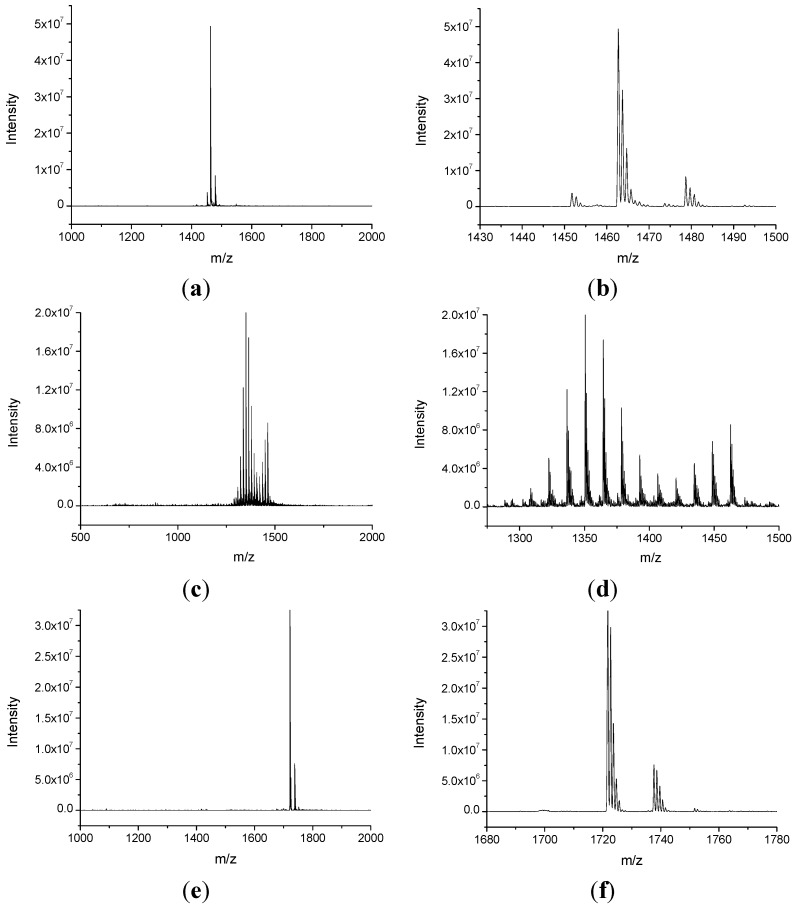

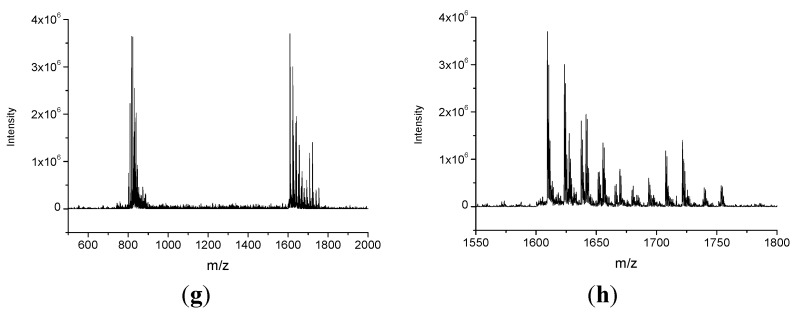
ESI-MS spectra in positive mode of **CD3** (**a**,**b**); **CD4** (**c**,**d**); **CD5** (**e**,**f**); **CD6** (**g**,**h**).

The synthesis of two βCD derivatives at different degrees of methylation (**CD5** and **CD6**) and bearing an NHPI moiety is outlined in Scheme 2. The methylation of **CD2**, the mono-azide-βCD derivative, with two different amounts of methyl iodide, as described in the Experimental Section, afforded **CD3** (permethylated) and **CD4** (random methylated). Both molecules were characterized by ^1^H-NMR, FT-IR (see Supplementary Materials for the spectra) and ESI-MS spectroscopies (see Figure 1a,b for **CD3** and Figure 1c,d for **CD4**). In particular, the ESI-MS spectrum in the positive mode of **CD3** shows the presence of peaks at *m*/*z* 1462.7 and *m*/*z* 1478.7 associated to [**CD3** + Na]^+^ and [**CD3** + K]^+^, respectively.

The ESI-MS spectrum of **CD4** is constituted by a more complex pattern of peaks in the range of *m*/*z* 1250–1500. In particular, it shows the peak at *m*/*z* 1462.7 corresponding to the sodium adduct of the fully methylated product (analogous to **CD3**), and a sequence of peaks associated to the sodium adducts of the products with a lower number of methyl groups. Further details on the peaks distribution are reported in Table 1 of SI. The calculation of the average molecular weight (${\bar{M}}_{W}$) and the *DS* of **CD4** were performed using Equations (SI1) and (SI2) in the Supplementary Materials providing the following values: ${\bar{M}}_{W}=1355\;g\;mol^{-1}$ and *DS* = *2.0*. A similar *DS* value (*1.91*) was obtained from the ^1^H-NMR spectrum of **CD4** in D_2_O.

**Table 1:** Methyl linoleate oxidation under O_2_ (1 atm) at 28 °C. Conditions: 5 mmol of methyl linoleate, catalyst (2% mol), 1.5 mL acetonitrile, 24 h.

Entry	Catalyst	Conversion ^1^ (%)	Selectivity ^2^ (%)	
			TC	TT
1		5.4	62	38
2	NHPI	42.7	49.5	50.5
3	CD5	54.7	50.6	49.4
4	CD6	48.0	53.8	46.2

^1^ See Equation (S3) in the Supplementary Materials. ^2^ See Equation (S4) in the Supplementary Materials.

The click reaction between **CD3** or **CD4** with the derivative **1** (*but-3-yn-1-yl 2-hydroxy-1,3-dioxoisoindoline-5-carboxylate*) to afford **CD5** and **CD6**, respectively, was carried out in THF as solvent in the presence of l-ascorbic acid/Cu^2+^ catalyst at room temperature and under a nitrogen atmosphere. The complete disappearance of the azide peak at 2102 cm^−1^ in the FT-IR spectra of both the derivatives (see Figure S12) was obtained in 48 h of reaction time. The FT-IR spectra of **CD5** and **CD6** confirmed the presence of a peak at 1734 cm^−1^, likely associated to the C=O stretching of the ester and the imide groups. The ^1^H-NMR spectrum of **CD5** obtained in acetone-*d*_6_ confirmed the presence of four peaks in the range between 7.6 and 8.6 ppm. Three of them are attributed to the NHPI aromatic ring protons (7.98 ppm, d, 1H; 8.33, s, 1H; 8.44 ppm, d, 1H) and one is associated to the proton of the triazole ring (7.86 ppm, s, 1H). The NO–H proton is visible as broad singlet at 9.92 ppm. The anomeric protons give a multiplet in the range 5.05–5.40 ppm (7H) while the H-6′ protons (2H) give a doublet of doublets in the range of 4.90–5.00 ppm. The triplet at 4.68 ppm is attributed to the CH_2_ adjacent to the ester group. A complex pattern of overlapping peaks in the range 2.90–4.20 ppm comprises the signals associated to the protons of the anhydroglucose units in the positions 2, 3, 4, 5 and 6, the CH_3_ signals and the signal of CH_2_ adjacent to the triazole ring (102H in total). The ESI-MS spectrum of **CD5** (Figure 1e,f) shows the presence of an intense peak at *m*/*z* 1721.7 associated to [**CD5** + Na]^+^ with the presence of two smaller peaks at *m*/*z* 1699.7 ([**CD5** + H]^+^) and *m*/*z* 1737.7 ([**CD5** + K]^+^). The ^1^H-NMR spectrum of **CD6** was obtained in DMSO-*d*_6_ with the addition of few drops of D_2_O in order to simplify its interpretation, which is similar to that of **CD5**.

Interestingly, the ESI-MS spectrum of **CD6** shows the presence of two sets of peaks (in the ranges *m*/*z* 1700–1780 and *m*/*z* 800–900). The detailed analysis of the first set of peaks (not reported) evidences the signals associated to the single charged sodium adducts of products at different level of methylation, with a distribution similar to the one of **CD4**. The second set of peaks is, instead, ascribed to the doubled charged sodium adducts. For comparison, the ESI-MS of **CD6** in negative mode (see Figure S11) shows the presence of only one set of peaks in the range *m*/*z* 1520–1710 attributed to [**CD6** − H]^−^ at different levels of methylation. The average molecular weight of **CD6** (1614 g·mol^−1^) was calculated from the one of **CD4**.

The two derivatives, **CD5** and **CD6**, have significantly different water solubilities. **CD6** is very soluble in water. At 28 °C, 71.6 mg of **CD6** were completely dissolved in only 76.5 mg of water obtaining a transparent viscous liquid. Due to the increased viscosity of the solution, no further addition of **CD6** was carried out. Therefore, **CD6** solubility is expected to exceed 950 g·L^−1^, a value significantly higher than that related to the solubility in water of simple NHPI (50.5 g·L^−1^) [32]. On the contrary, **CD5** has a lower, but not negligible, solubility, as expected on the basis of its molecular structure. In fact, only 16.0 mg could be dissolved into 860 mg of water, leading to a solubility value of just 18.8 g·L^−1^ at 28 °C. These results confirm the versatility of the proposed systems, as it is possible to modulate the hydrophilicity/hydrophobicity of the organocatalysts by monitoring the methylation of the cavitand. This aspect has direct effects on the solubility of the NHPI derivative, but also allows to better-fit the structure chemistry for a high permeability of the cellular membrane by the supramolecular pro-oxidant.

### 2.2. Catalytic Activity

PUFA, the essential components of cellular membranes, are particularly prone to peroxidation, and, for this reason, methyl linoleate has been chosen as a representative substrate for testing the catalytic activity of **CD5** and **CD6** pro-oxidants. The high reactivity of these lipids towards oxygen is associated to the low bond dissociation energy (BDE) of the C–H bonds in the bis-allylic position (~73 Kcal/mol) [33], which favors hydrogen atom abstraction from peroxyl radicals. Primary products of peroxidation are two trans/cis hydroperoxides, deriving from the hydrogen substitution with oxygen at the 13 and 9 positions, and two trans/trans analogues, with a more stable geometry, formed via β-fragmentation of the intermediate peroxyl radicals (Scheme 1). In the absence of hydrogen donors, the mechanism may also include fragmentation and cyclization reactions, leading to the formation of secondary products.

In the presence of NHPI, the reaction run faster and with higher selectivity in hydroperoxides [23]. In fact, hydrogen atom abstraction from bis-allylic position by PINO radical is thermodynamically favored, being that the O–H BDE value in the corresponding NHPI is significantly higher (88.1 Kcal/mol) [34]. Moreover, NHPI traps peroxyl radicals before they undergo secondary reactions, limiting the formation of products uniquely to hydroperoxides (Scheme 1).

The methyl linoleate peroxidation experiments were carried out using **CD5** or **CD6** as catalysts, in the absence of an initiator and in presence of the minimum amount of acetonitrile required for obtaining a homogeneous mixture. For comparison, the same experiments were also carried out using NHPI. The identification of the peroxidation products and their quantification were obtained by ^1^H-NMR spectroscopy, following the paper of Pajunen *et al.* [35]. This technique is simple, fast, and reliable, and represents a valid alternative to the more cumbersome HPLC analysis protocol [23]. Further details can be found in the Supplementary Materials.

**Figure 2:**
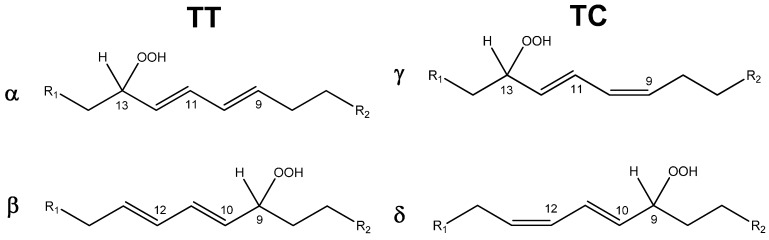
Different hydroperoxides formed during the aerobic oxidation of methyl linoleate catalyzed by CD-NHPI derivatives. **α**: Methyl 13-(*R*,*S*)-hydroperoxy-9-*trans*,11-*trans*-octadecadienoate; **β**: Methyl 9-(*R*,*S*)-hydroperoxy-10-*trans*,12-*trans*-octadecadienoate; **γ**: Methyl 13-(*R*,*S*)-hydroperoxy-9-*cis*,11-*trans*-octadecadienoate; **δ**: Methyl 9-(*R*,*S*)-hydroperoxy-10-*trans*,12-*cis-*octadecadienoate. R_1_ = CH_3_(CH_2_)_3_; R_2_ = (CH_2_)_5_CO_2_CH_3_.

Different from HPLC analysis, ^1^H-NMR spectroscopy does not allow to distinguish the hydroperoxides **α** and **β** or the hydroperoxides **γ** and **δ** (see Figure 2). Therefore, **α** and **β** will be simply identified as “trans-trans” (**TT**) hydroperoxides, while **γ** and **δ** will be identified as “trans-cis” (**TC**) hydroperoxides. However, this aspect is not crucial for the scope of this work. Indeed, following the initial motivation of the work, oriented to the proposal of new pro-oxidant catalysts, the scope of the paper is aimed at demonstrating the effective capability of **CD5** and **CD6** in catalyzing a lipid peroxidation process without any particular interest for the regio- and stereoselectivity. Table 1 summarizes the experimental results, providing the methyl linoleate conversion and the selectivity of the oxidative process to **TC** and **TT** hydroperoxides. In the absence of catalyst, the autoxidation process led to a very low conversion (5.4%, entry 1). In the presence of **CD5** (entry 3) and **CD6** (entry 4), the conversions were significantly higher and comparable with the value associated to NHPI (entry 2).

## 3. Experimental Section

### 3.1. General

β-cyclodextrin (βCD) and other reagents and solvents were commercially available and used as received unless otherwise stated. 6A-*O*-p-toluenesulfonyl-βCD (**CD1**) was obtained according to a published procedure [36] with minor modifications. The corresponding monoazide derivative (**CD2**) was obtained reacting **CD1** with an excess of NaN_3_ in DMSO at 90 °C overnight. The methylation of **CD2** in order to obtain the derivatives **CD3** and **CD4** was performed in anhydrous DMF (60 mL) following the procedure similar to the one described by Muderawan *et al.* [37]. All the details are reported in the Supplementary Materials.

^1^H-NMR spectra of the products were recorded at 305 K with a Bruker Avance-500 MHz NMR spectrometer (Bruker, Billerica, MA, USA). The FT-IR solid phase spectra of the powdered sample with infrared grade KBr were recorded using a Varian 640-IR spectrometer (Varian, Palo Alto, CA, USA). ESI-MS mass spectra were collected on a Bruker Esquire 3000+ with electrospray ionization source and ion-trap detector. The samples were analyzed by direct infusion of suitable solutions (methanol) in the spectrometer source.

### 3.2. Synthesis of the Derivative ***1*** (But-3-yn-1-yl 2-hydroxy-1,3-dioxoisoindoline-5-carboxylate)

Trimellitic anhydride chloride (4.21 g, 20 mmol) was dissolved in 40 mL of anhydrous THF with 3 mL of anhydrous pyridine. Then, 20 mL of a solution of 3-butyn-1-ol (1.40 g, 20 mmol) in anhydrous THF were added dropwise over 1 h under stirring at 0 °C. The mixture was kept at 0 °C for 6 h and then at room temperature overnight. The white precipitate formed during the reaction was removed by filtration on paper while the solution was concentrated under vacuum. An excess of hydroxylamine hydrochloride (30 mmol, 2.09 g) was added to the thus-obtained syrup after diluting it with 25 mL of anhydrous pyridine. The mixture was stirred at room temperature for 30 min and then under reflux for 1 h in a microwave reactor with the automatic control of the temperature (Micro-SYNTH Labstation-Milestone Inc., Shelton, CT, USA). The solvent was removed under vacuum, obtaining a bright orange sticky solid that was finally washed with HCl (0.1 M) in a ice bath until complete removal of the residual pyridine. The procedure provided, in quantitative yield, an off-white solid that was used for the preparation of **CD5** and **CD6** without further purification. ^1^H-NMR (400 MHz, DMSO-*d*_6_): δ = 10.95 (s, 1H), 8.36 (d, 1H), 8.20 (s, 1H), 7.98 (d, 1H), 4.41 (t, 2H, *J* = 6.57 Hz), 2.86 (t, 1H, *J* = 2.69 Hz), 2.704 (td, 2H, *J*_1_ = 6.57 Hz, *J*_2_ = 2.69 Hz), ^13^C-NMR (100 MHz, DMSO-*d*_6_): 164.42, 163.62, 163.60, 135.70, 135.13, 133.05, 129.79, 123.87, 123.15, 81.14, 72.95, 63.81, 18.77.

### 3.3. Synthesis of ***CD5*** Derivative

**CD3** (1.86g, 1.29 mmol) and **1** (670 mg, 2.58 mmol) were dissolved in 25 mL of THF under slow N_2_ flow. Then, 1 mL of an aqueous solution of l-ascorbic acid (88.1 mg, 0.5 mmol), CuSO_4_ × 5H_2_O (62.4 mg, 0.25 mmol) and Et_3_N (2 droplets) were added to the reaction mixture. The system was stirred at room temperature for 48 h under N_2_ atmosphere. After evaporating the solvent, the solid was transferred into a separatory funnel and extracted with EtOAc (3 × 100 mL). The organic phases, containing both **CD5** and the excess of **1**, were collected together, dried onto anhydrous Na_2_SO_4_ and concentrated under vacuum. The corresponding syrup was transferred onto a silica gel column and eluted with Et_2_O. After the complete removal of the derivative **1**, the product **CD5** was collected by eluting the column with a CHCl_3_:MeOH (10:1) solution (about 300 mL). Finally, the orange colored organic phase was washed with HCl 0.1 M in a separatory funnel, dried onto anhydrous Na_2_SO_4_ and evaporated under vacuum, obtaining a pale yellow solid (2.05 g, ≈93.5% yield).

### 3.4. Synthesis of ***CD6*** Derivative

**CD6** was obtained following a procedure similar to **CD5** from 678 mg of **CD4** (0.5 mmol) and 520 mg (2.00 mmol) of **1** dissolved in 15 mL of THF. The purification on silica gel column was carried out, first eluting with Et_2_O in order to remove the residual **1**, and then with MeOH to recover the product. MeOH was almost completely evaporated under vacuum. The mixture was diluted in CHCl_3_ (150 mL) and washed with HCl 0.1 M in a separatory funnel. The organic phase was dried onto anhydrous Na_2_SO_4_ and finally evaporated under vacuum, recovering a pale yellow solid (820 mg, 50.8%).

### 3.5. General Procedure for the Methyl Linoleate Aerobic Peroxidation

The catalyst (**CD5** (172.2 mg, 0.1 mmol) or **CD6** (161.4 mg, 0.1 mmol) or NHPI (16.3 mg, 0.1 mmol)) was dissolved in 1.5 mL of acetonitrile with 5 mmol (1.472 g) of methyl linoleate. The solution was stirred under O_2_ (1 atm) at 28 °C for 24 h. Three hundred microliters of this solution were loaded onto a short silica gel column and eluted with hexane:ethyl acetate 1:1 (about 3 mL) in order to remove the catalyst. The eluent was evaporated under flux of nitrogen in about 1 h at 28 °C obtaining a transparent oil that was analyzed by ^1^H-NMR spectroscopy. Further details on the characterization are reported in the Supplementary Materials.

## 4. Conclusions

The synthesis of two new supramolecular pro-oxidant organocatalysts, **CD5** and **CD6**, has been reported. The synthetic approach is based on the functionalization of permethylated and random methylated cyclodextrins with NHPI moieties by a click chemistry approach. Final products, fully characterized by NMR, IR and ESI-MS spectroscopic techniques, showed a completely different solubility in water medium, revealing how the cyclodextrin methylation degree can be an ideal tool to modulate catalyst solubility. Moreover, the linking of NHPI to methylated cyclodextrin cavitands, which have a higher affinity with cellular membranes, opens the route for the use of these catalysts as possible pro-oxidant agents in cancer therapy. For this reason, the catalytic activity of the new systems has been successfully verified in the oxidation of methyl linoleate, chosen as a representative sample as the corresponding cholesteryl ester as a key component of lipid membranes. Both the catalysts showed a catalytic efficiency analogous to that of simple NHPI, confirming how cyclodextrin functionalization does not affect NHPI reactivity. As expected, the oxidation process is selective in the formation of the corresponding hydroperoxides, without leading to the formation of secondary products. Further studies will be conducted in order to verify the possible catalytic activity of these systems in biological mediums.

## Supplementary Materials

Supplementary materials can be accessed at: http://www.mdpi.com/1420-3049/20/09/15881/s1.
