# Supplementary material for: Functionalization of Cyclodextrins with N-Hydroxyphthalimide Moiety: A New Class of Supramolecular Pro-Oxidant Organocatalysts
Source: Molecules. 2015 Aug 31;20(9):15881–92. doi: 10.3390/molecules200915881 (PMC6332168; doi:10.3390/molecules200915881)
Supplement: Supplementary file 1 [file molecules-20-15881-s001.pdf]

Supplementary File

Synthetic procedure of **CD1**	S1
Synthetic procedure of **CD2**	S1
^{1}H-NMR and ESI-MS of **CD1**	S2
^{1}H-NMR and ESI-MS of **CD2**	S3
^{1}H-NMR and ^{13}C-NMR of **1**	S4
^{1}H-NMR of **CD3** and **CD5**	S5
^{1}H-NMR of **CD4** and **CD6**	S6
ESI-MS spectrum of **CD6** in negative mode	S7
FT-IR spectra of **CD3**, **CD4**, **CD5** and **CD6**	S8
Calculation of **CD4** molar mass from ESI-MS in positive mode	S8–S9
Oxidation of methyl linoleate: ^{1}H-NMR spectra	S9–S10

Synthesis of CD1

11.35 g (10 mmol) of β-CD were dissolved in 200 mL of 0.6 M NaOH(aq) at 0 °C. After 30 min, 2.3 g (12.1 mmol) of 4-Toluenesulfonyl chloride (TsCl), finely powdered with a mortar and pestle, were added to the aqueous solution. The reaction mixture was magnetically stirred for 8 h at 0 °C. The unreacted TsCl was removed by filtration onto a sintered glass funnel. The aqueous phase was acidified at 0 °C with 10 mL of HCl (37%) added dropwise. The product precipitated as white solid. The mixture was kept at 4 °C overnight, filtered on paper and washed with deionized water until neutral pH. In order to reduce the content of unreacted β-CD, the solid was dispersed in 100 mL of hot water (65 °C), stirred for 10 min, and finally filtered. This procedure was qualitatively followed by TLC (eluent: 2-propanol:H_2O:EtOAc:NH_4OH = 5:3:1:0.5) and repeated for 3 times until the β-CD spot on TLC (R_f: 0.25) was negligible compared to the **CD1** spot (R_f: 0.47). The final product was washed with acetone (100 mL × 3 times), dried in air for 24 h and under vacuum (<5 mbar) at 25 °C for 3 h. Yield: 35% (4.50 g). The tosylation procedure gave also the formation of very small amounts of ditosylates (R_f = 0.58), according to the literature [Brady2000]. Due to the small ΔR_f between the mono- and the di-tosylate no attempt of further separation was made. ESI-MS analysis performed on the final product confirmed the presence of negligible amounts of pristine CD and di-tosylate (see Figure SI2).

[Brady2000] Brady, B.; Lynam, N.; O'Sullivan, T.; Ahern, C.; Darcy, R. 6A-*O*-p-toluenesulfonyl-β-cyclodextrin, *Org. Synth.* **2000**, *77*, 225, doi:10.15227/orgsyn.077.0225.

Synthesis of CD2

3.48 g (3 mmol) of **CD1** were dissolved into 10 mL of DMSO. An excess of sodium azide (390 mg, 6 mmol) was added to the solution. The mixture was heated at 90 °C for 12 h. After cooling it at room temperature, the product was obtained by precipitation in acetone and filtering on filter paper. The white solid was washed with acetone (100 mL × 4 times) and dried in air. The excess of NaN_3 was removed by treating the aqueous solution containing the product (100 mL) with IRA900-Cl ionic exchange resin. Water was then removed under rotary evaporation. The white solid was finally washed with acetone (100 mL × 3 times) and dried in air. Quantitative yield.

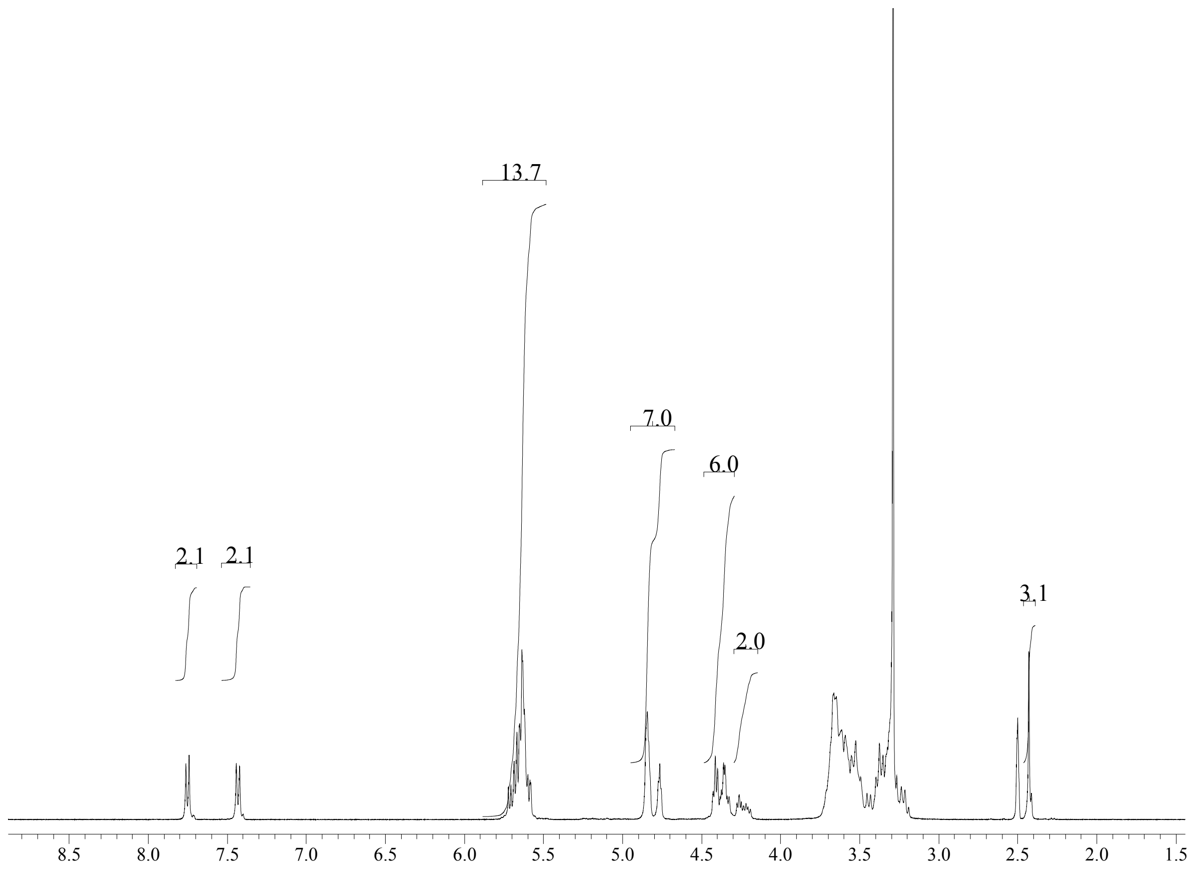

Figure S1. ^{1}H-NMR spectra of **CD1** in DMSO-d_6 (305 K).

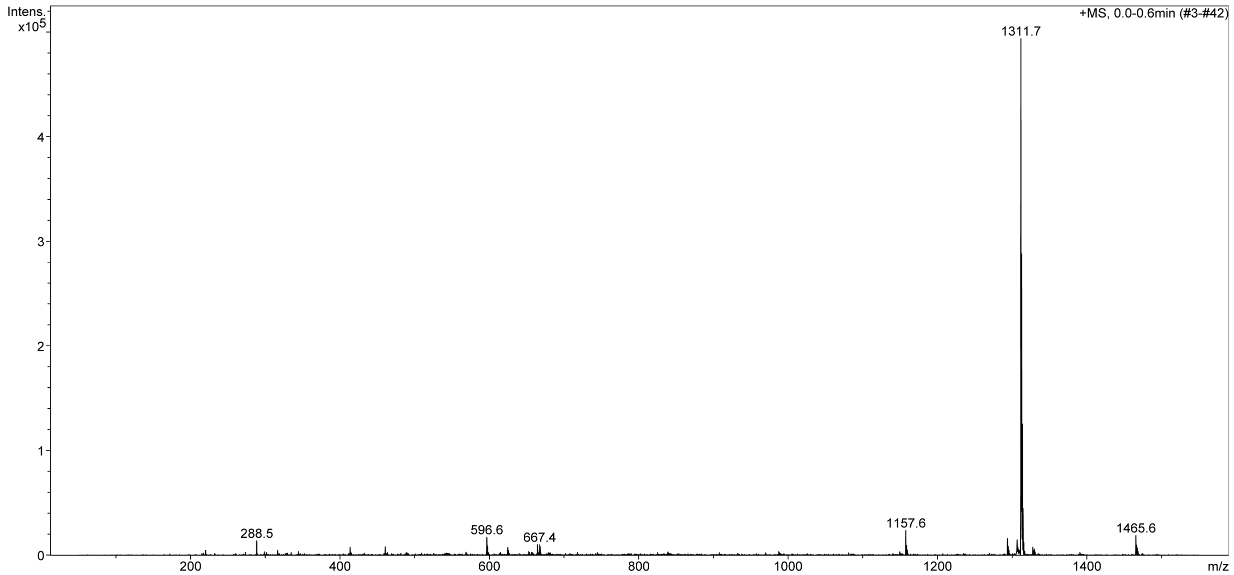

Figure S2. ESI-MS of **CD1**.

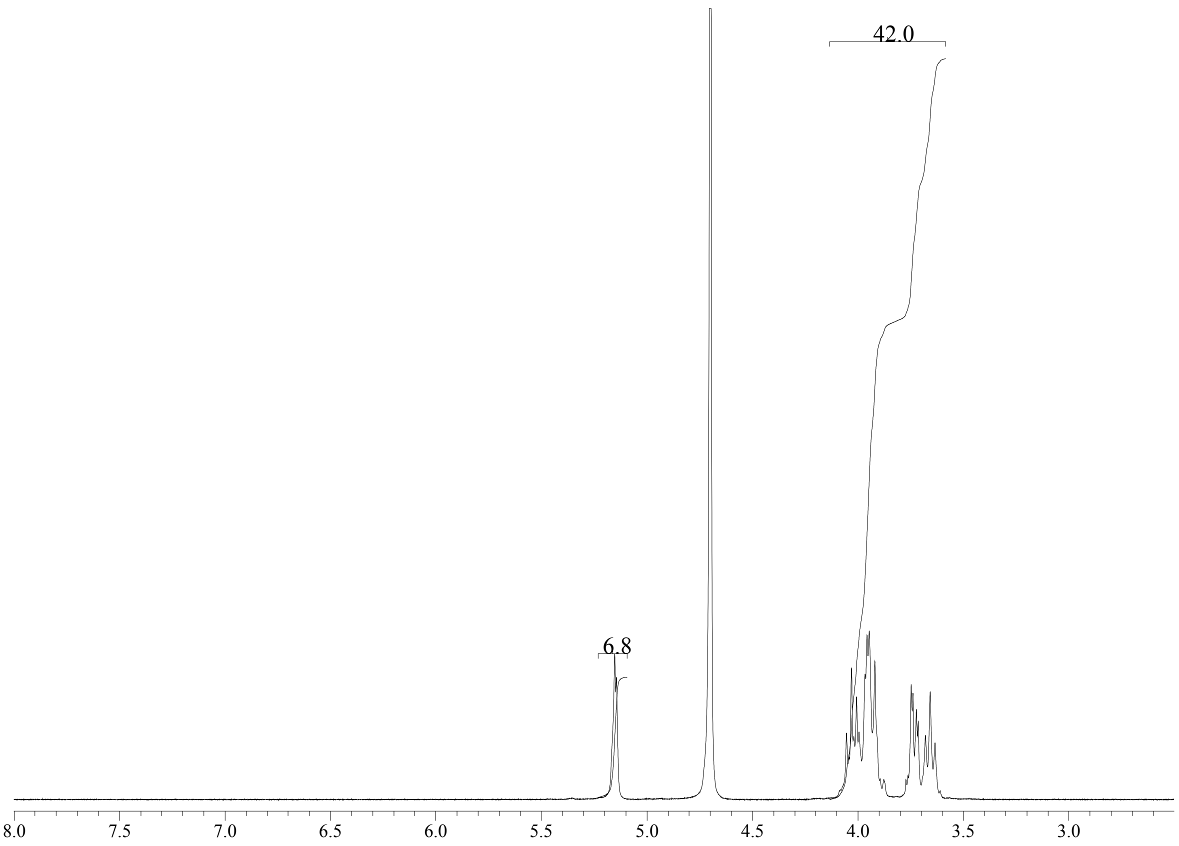

Figure S3. ^{1}H-NMR spectra of **CD2** in D_2O (305 K).

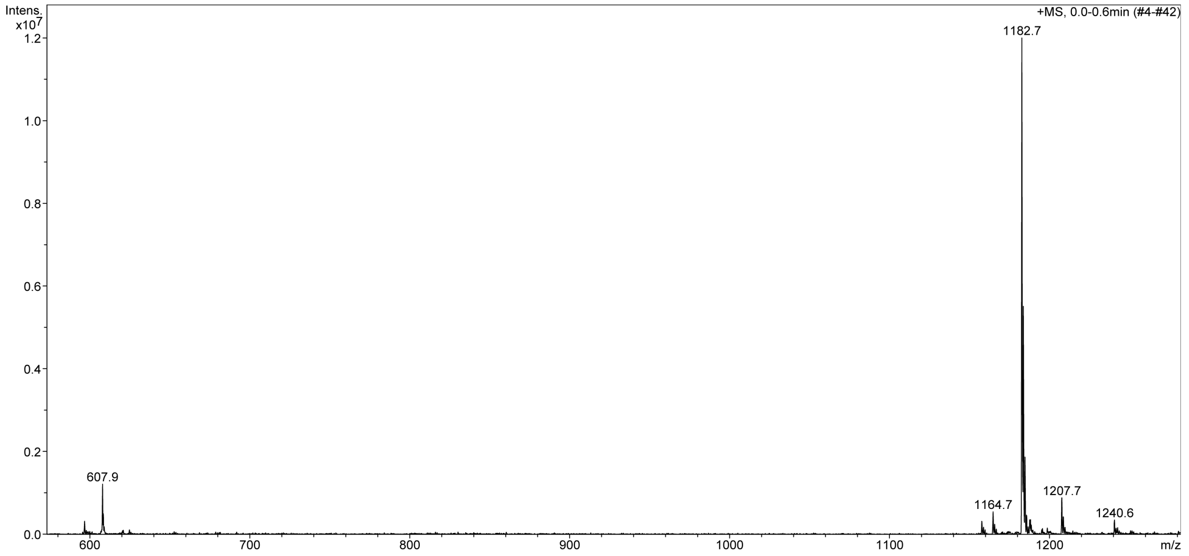

Figure S4. ESI-MS of **CD2**.

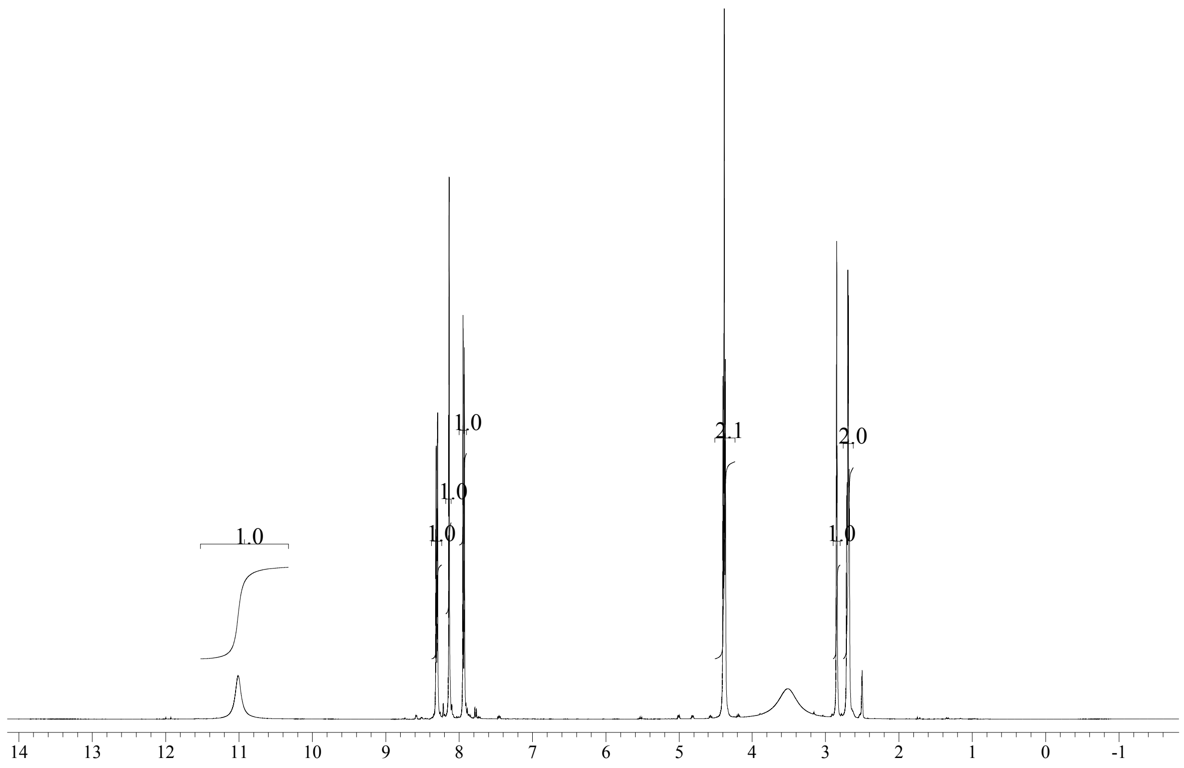

Figure S5. ^{1}H-NMR spectrum of **1** in DMSO-*d*$_6$.

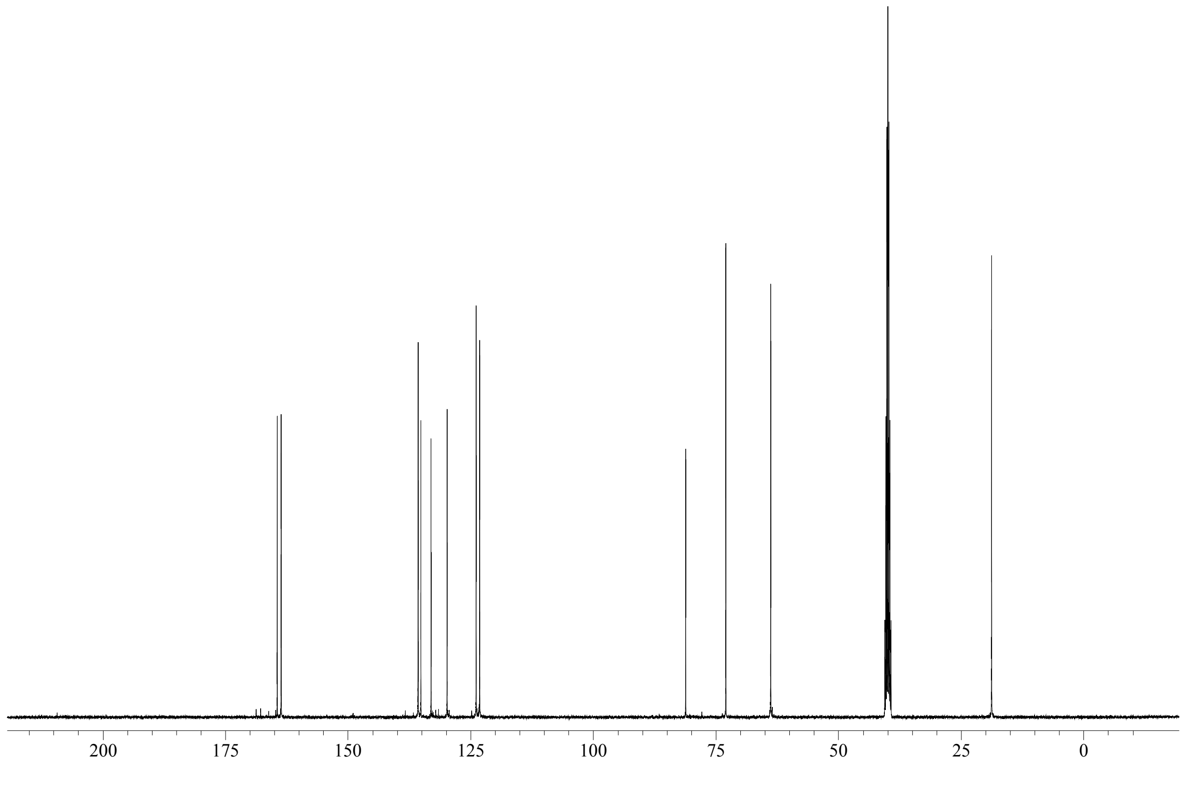

Figure S6. ^{13}C-NMR spectrum of **1** in DMSO-*d*$_6$.

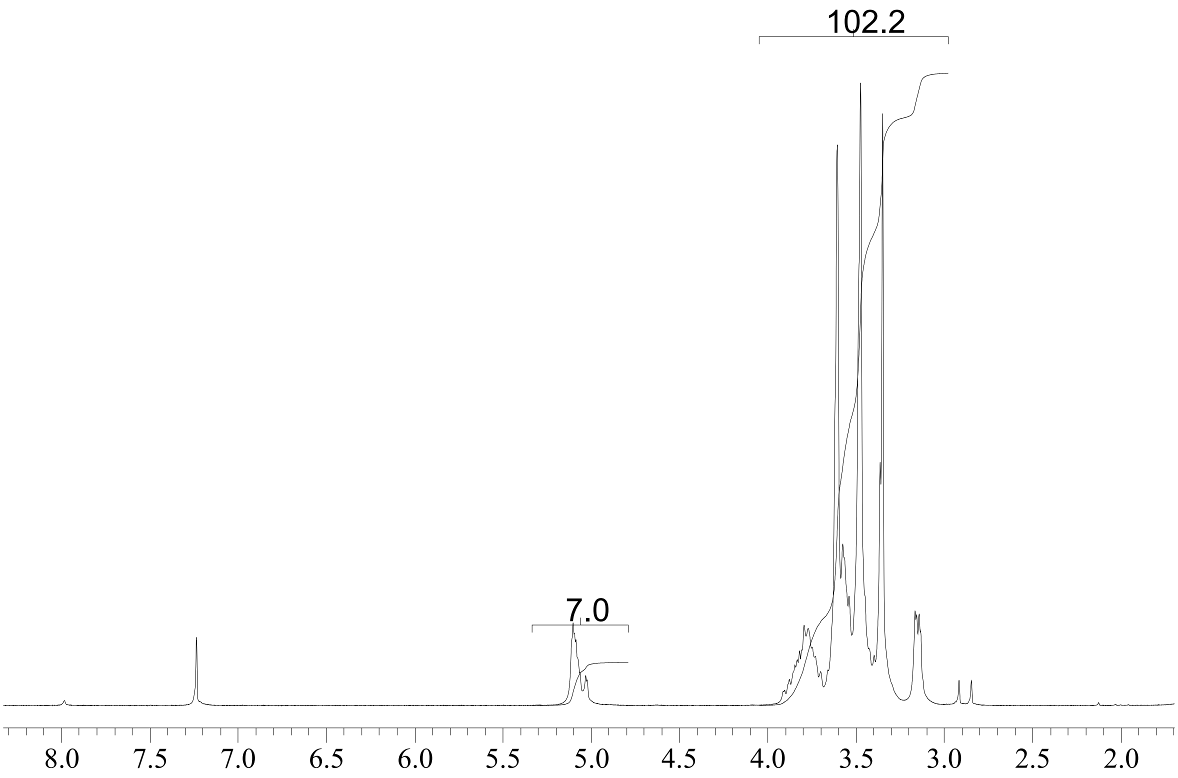

Figure S7. ^{1}H-NMR spectrum of **CD3** in $CDCl_3$.

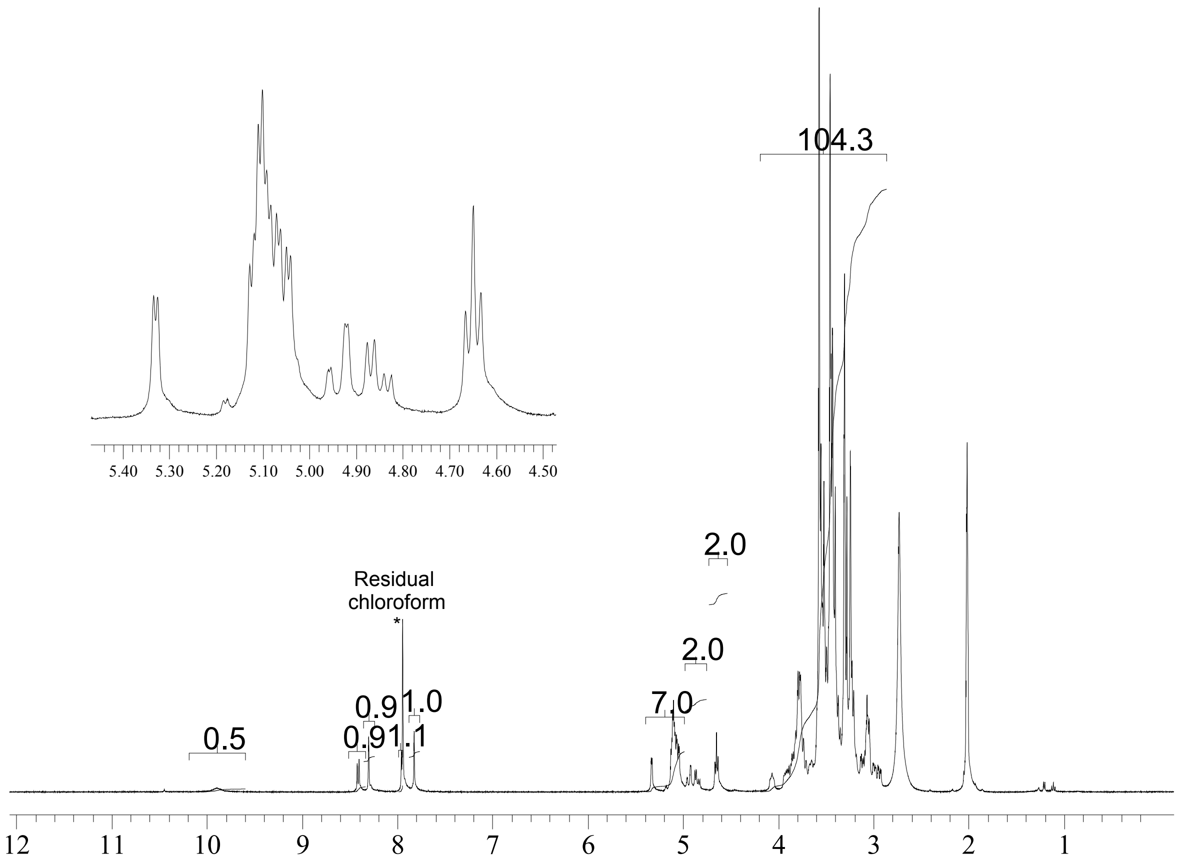

Figure S8. ^{1}H-NMR spectrum of **CD5** in acetone-d_6. The inset is a magnification of the signals in the range 4.50–5.50 ppm.

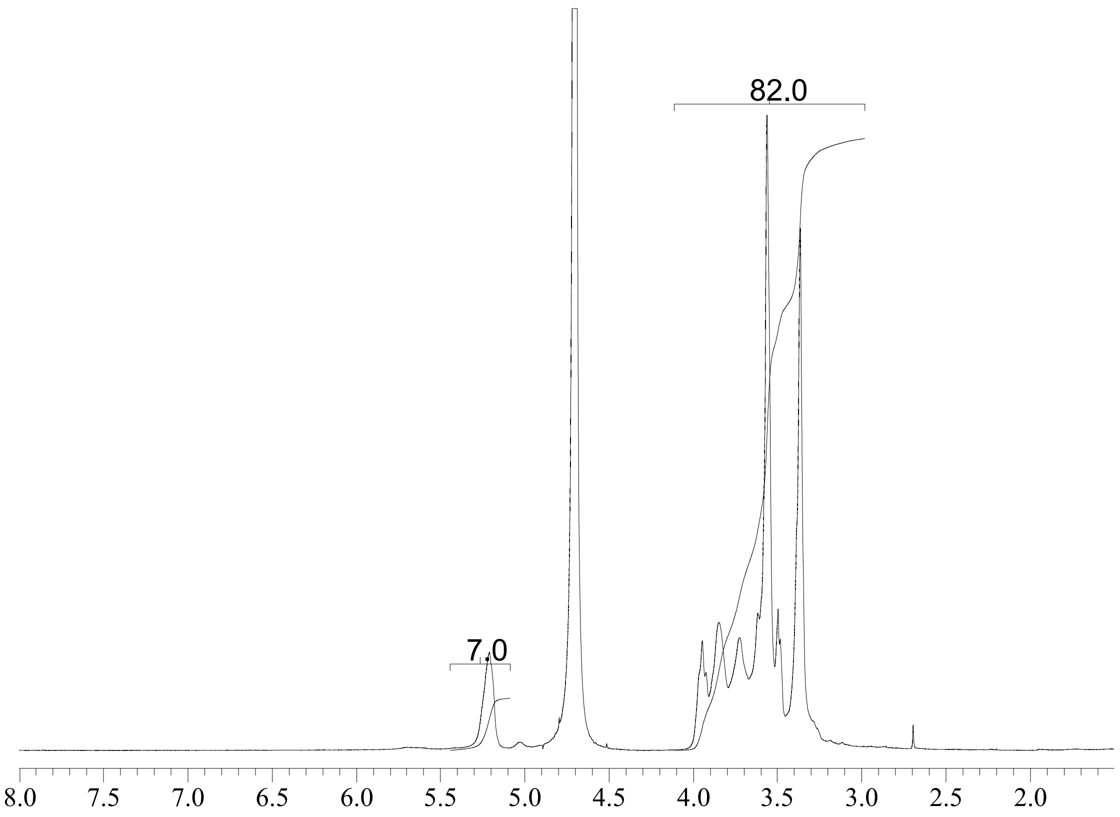

Figure S9. ^{1}H-NMR spectrum of **CD4** in D_2O.

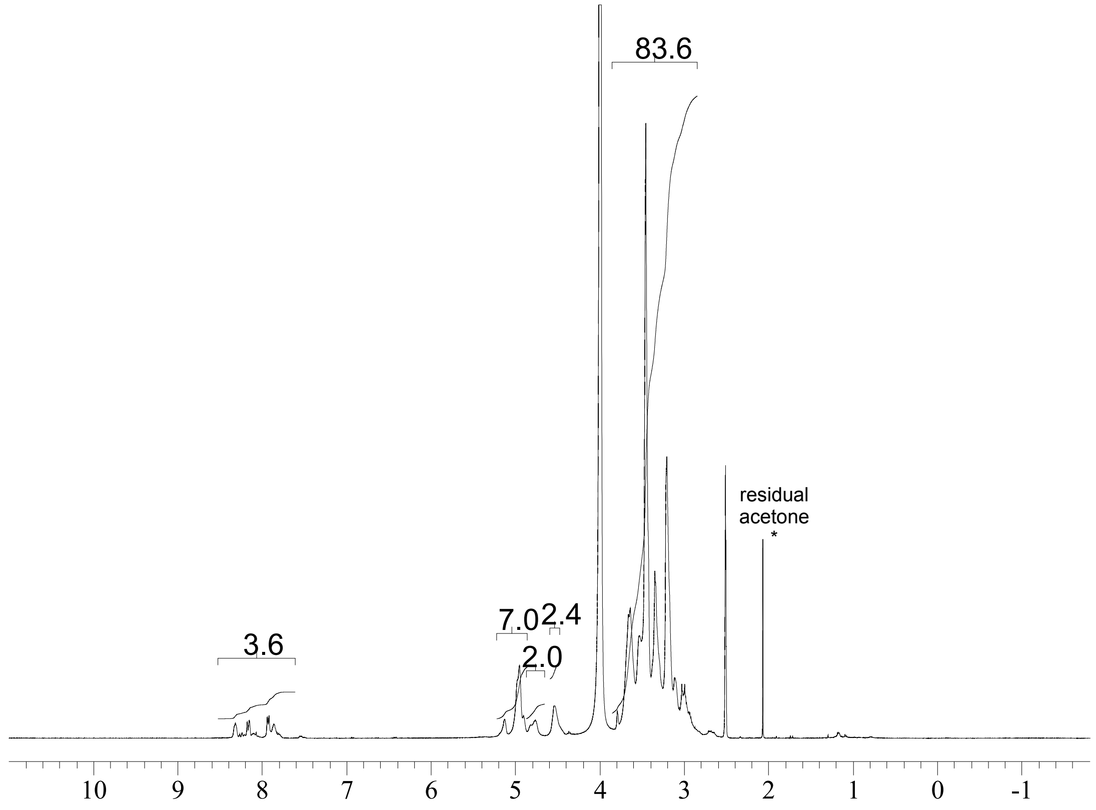

Figure S10. ^{1}H-NMR spectrum of **CD6** in DMSO-d_6 + D_2O.

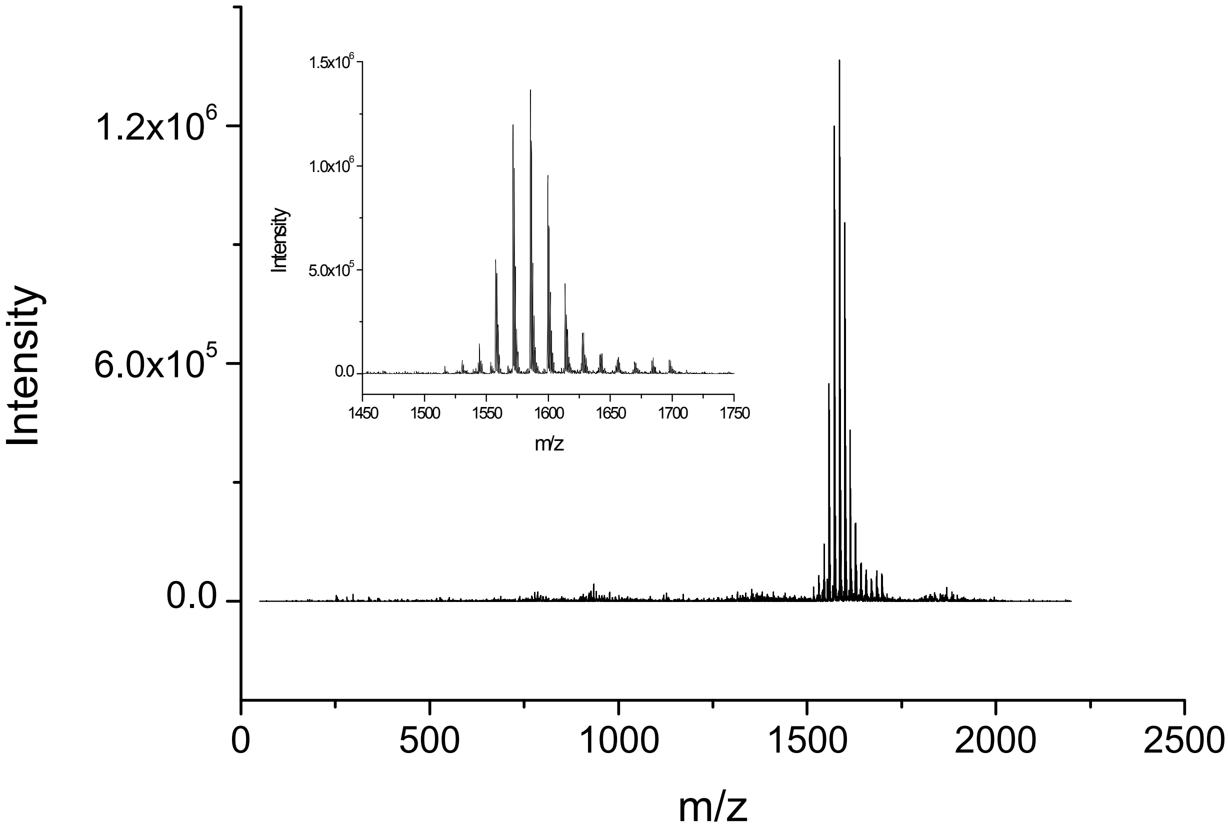

Figure S11. ESI-MS spectrum in negative mode of **CD6**. The inset shows a magnification of the peaks in the range *m*/*z* 1450–1750.

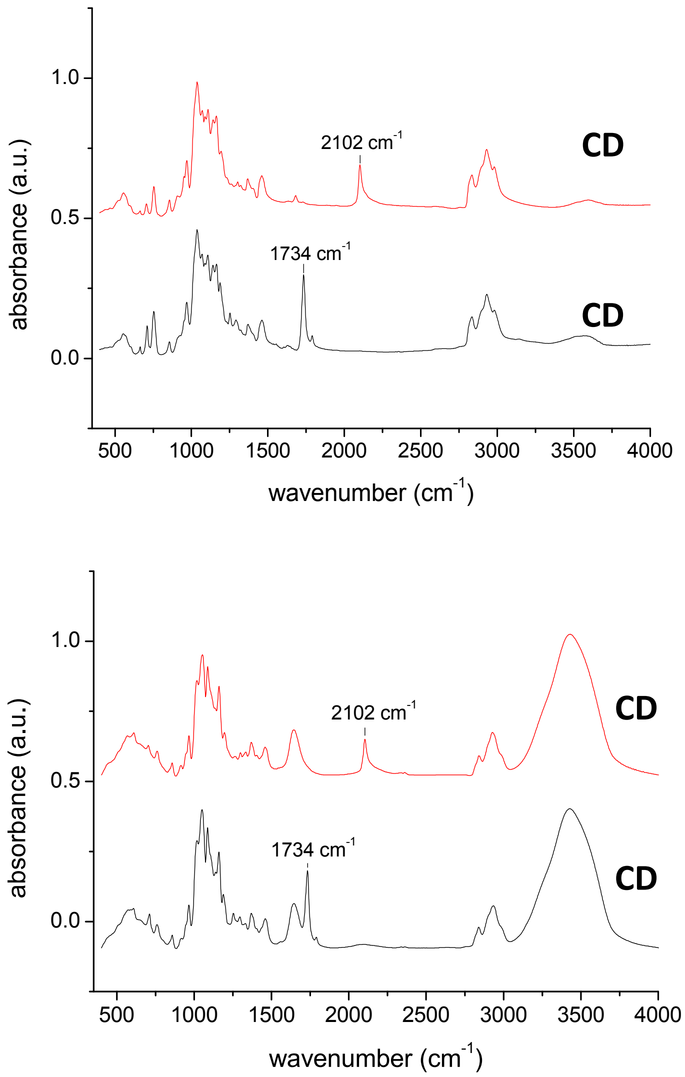

Figure S12. FT-IR spectra (KBr) of the different derivatives.

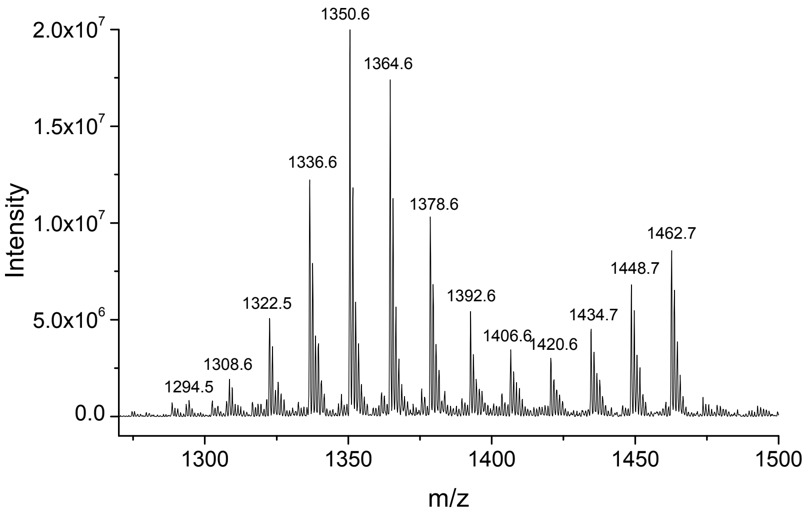

Figure S13. ESI-MS of **CD4** in positive mode. The peaks are associated to the sodium adducts of the derivatives at different level of methylation (see Table S1).

Table S1. ESI-MS of **CD4** in positive mode. Distribution of the peaks (sodium adducts).

**n.Me groups (n_{Me})**	**M_w (g·mol^{-1})**	**Intensity (I_{Me})**
**8**	1294.5	8.33×10^5
**9**	1308.6	1.93×10^6
**10**	1322.5	5.07×10^6
**11**	1336.6	1.22×10^7
**12**	1350.6	2.02×10^7
**13**	1364.6	1.74×10^7
**14**	1378.6	1.03×10^7
**15**	1392.6	5.42×10^6
**16**	1406.6	3.45×10^6
**17**	1420.6	3.02×10^6
**18**	1434.7	4.51×10^6
**19**	1448.7	6.81×10^6
**20**	1462.7	8.57×10^6

$$\overline{M}_W = \frac{\sum_{Me=8}^{20} M_W \cdot I_{Me}}{\sum_{Me=8}^{20} I_{Me}} - 23 \qquad \text{(S1)}$$

$$DS = \frac{1}{7} \cdot \frac{\sum_{Me=8}^{20} n_{Me} \cdot I_{Me}}{\sum_{Me=8}^{20} I_{Me}} \qquad \text{(S2)}$$

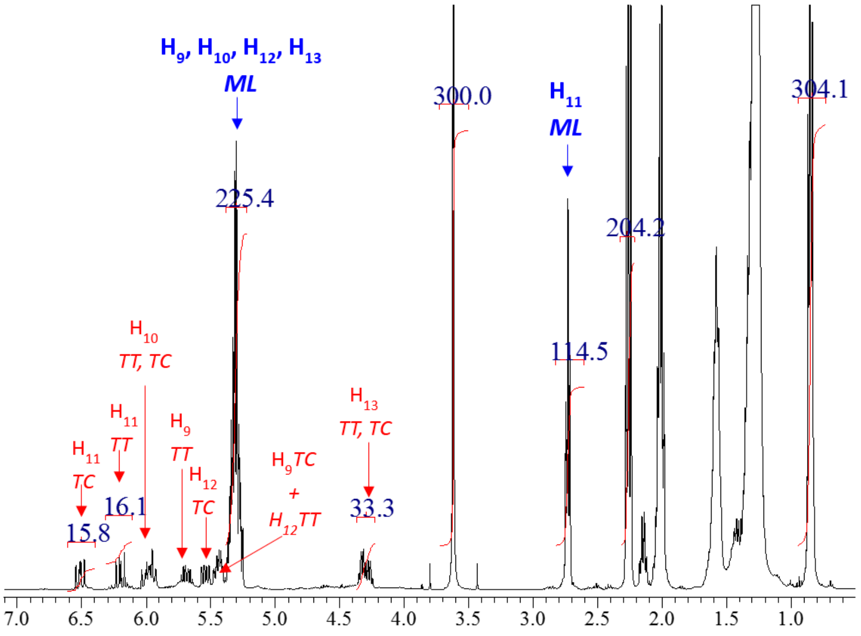

Figure S14. ^{1}H-NMR spectrum of methyl linoleate after oxidation under O_2 (1 atm) at 28 °C. Conditions: 5 mmol of methyl linoleate, NHPI (2% mol), 1.5 mL acetonitrile, 24 h. Assignment of the peaks according to the work of Pajunen *et al.*, *Chem. Phys. Lipids* **2008**, *154*, 105–114. (Ref. 35 in the manuscript).

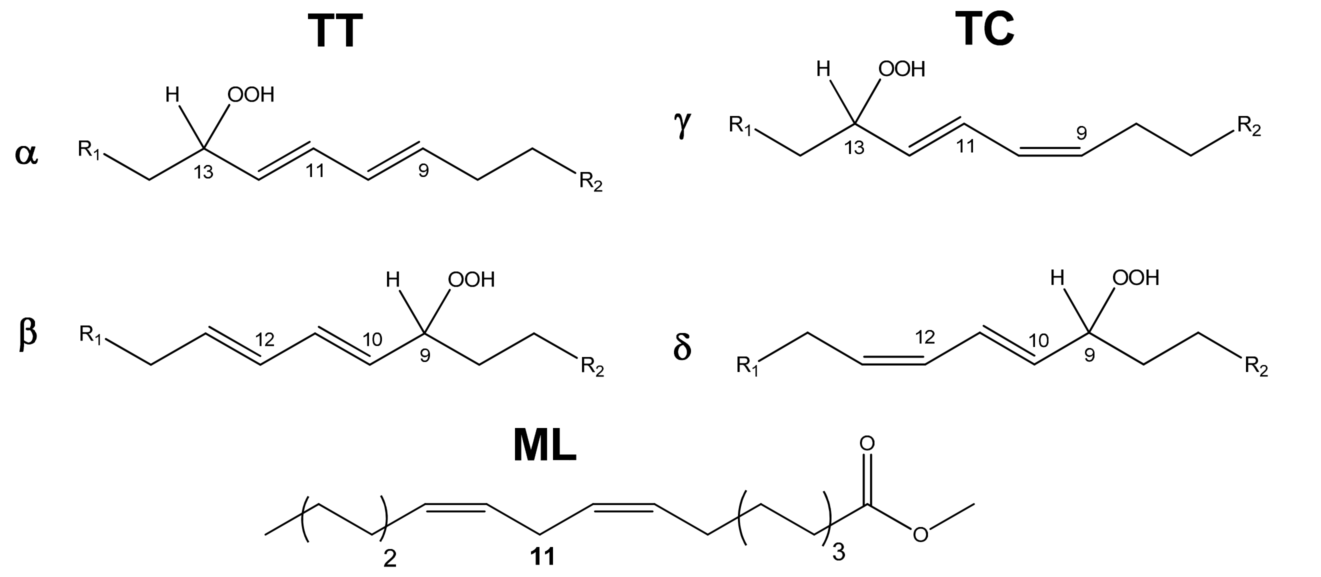

Figure S15. Molecular structures. **ML**: methyl linoleate; **TC**: "trans-cis" hydroperoxides; **TT**: "trans-trans" hydroperoxides.

ML conversion can be calculated from the ^{1}H-NMR spectrum of the oxidized methyl linoleate (Figure S14) with the following Equation (SI3) considering the area of the peak associated to the olefinic proton of the residual methyl linoleate (H_9, H_{10}, H_{12} and H_{13}-5.24–5.37 ppm):

$$\mathrm{Conv} = 100 \times \frac{400 - \mathrm{H_n}(ML)}{400} \quad n = 9,10,12,13 \tag{S3}$$

The selectivity in TC and TT was obtained from the areas of the peaks of H_{11} TC (6.40–6.60 ppm) and H_{11} TT (6.10–6.30 ppm):

$$\mathrm{Sel(TC))} = 100 \times \frac{\mathrm{H_{11}}(TC)}{H_{11}(TC) + \mathrm{H_{11}}(TT)} \tag{S4}$$
